# Pathological Mechanisms Involved in HIV-Associated Lymphomagenesis: Novel Targeted Therapeutic Approaches

**DOI:** 10.3390/cells14100705

**Published:** 2025-05-13

**Authors:** Mihaela Straista, Francesca Caccuri, Nicoleta Arnaut, Arnaldo Caruso, Mark Slevin

**Affiliations:** 1 Centre for Advanced Medical and Pharmaceutical Research, “George Emil Palade” University of Medicine, Pharmacy, Science and Technology, 540142 Targu Mures, Romania; strastimira@gmail.com (M.S.); francesca.caccuri@unibs.it (F.C.); arnaut.nicoleta99@gmail.com (N.A.); mark.slevin@umfst.ro (M.S.); 2 Section of Microbiology, Department of Molecular and Translational Medicine, University of Brescia, 25123 Brescia, Italy

**Keywords:** HIV, lymphomagenesis, vp17, therapeutic approaches, chronic B cell activation

## Abstract

The intricate interplay of direct and indirect mechanisms relating to immune dysfunction, chronic inflammation, and viral proteins represents a key factor of lymphomagenesis in HIV-infected patients. Indirect mechanisms based on cytokine dysregulation, HIV-induced immune dysfunction, and co-infections with oncogenic viruses induce chronic B-cell activation and generation of a prone environment for malignant transformation and tumor growth. Direct mechanisms arise from oncogenic influences of p17, Tat, and Nef HIV proteins, which generate genomic instability, alteration of cellular signaling, and activation of oncogenic pathways. Vp17’s implication in lymphomagenesis and angiogenesis, ensured by activation of PAR1/EGFR/PI3K/Akt and MEK/ERK1/2 pathways, emphasizes the critical need for developing therapeutic strategies that target their signaling mechanisms. This review shows an insight into the pathological mechanisms involved in lymphomagenesis in HIV-infected individuals, focusing on finding novel therapeutic approaches directed at immune rehabilitation and oncogenic signaling pathways.

## 1. Introduction

HIV-associated cancers can be classified into two groups—acquired immune deficiency syndrome (AIDS) defining and non-AIDS-defining cancers. On the list of AIDS-defining cancers are Kaposi’s sarcoma (KS), cervical cancer, and non-Hodgkin lymphoma (NHL). Other cancers like anal cancer, lung cancer, vulvar cancer, penile cancer, hepatocellular cancer, and Hodgkin’s lymphoma (HL) are included on the non-AIDS-defining cancers list [1]. The wider use of combined antiretroviral therapies (cART) altered the epidemiology of HIV-associated cancers due to an incomplete immune recovery. Compared to the general population, the risk of NHL and HL in PLWHIV remains elevated, and this may be attributed to increased survival in the cART era, resulting in an aging HIV-positive population and a corresponding rise in cancer burden [2]. HIV-associated lymphomas (HALs) are the most common malignancy among them [3]. The main subtypes are Burkitt’s lymphoma (BL), diffuse large B-cell lymphoma (DLBCL), and HL [4].

Current standard treatment used for HALs implies an association between cART and chemotherapy (ChT), however, even though cART may improve ChT tolerability and enhance immune reconstitution, potential pharmacodynamic and pharmacokinetic interactions between them might appear. Moreover, their efficiency in refractory or relapsed lymphoma subtypes like DLBCL or BL has not been demonstrated because of a lack of clinical trials data available to date [4]. This emphasizes the need to develop new therapeutic strategies that will target other mechanisms involved in lymphomagenesis regarding PLWHIV.

Various mechanisms have been proposed to explain B-cell transformation in HIV-positive patients that leads to HALs. The pathogenesis of HALs is based on both indirect and direct pathways involving HIV proteins, cytokines, impaired macrophages, T cells, and co-infections. These mechanisms, taken together, induce immune dysfunction, genomic instability, and lymphomagenesis [5]. Gaining insight into these mechanisms is crucial for developing new therapeutic strategies that will target precisely the mechanism, or the molecules involved. The purpose of this study is to identify the main mechanisms involved in HIV lymphomagenesis and use them as potential targets for novel treatments.

## 2. Main Text

### 2.1. Chronic Inflammation Is Proposed as an Initiator of the Indirect Pathway

The first target of HIV is the gut, whose disruption leads to dysbiosis and microbial translocation. This discharge of bacterial products into the bloodstream generates inflammation and causes chronic immune activation [6,7]. A prospective study by Epeldegui et al. demonstrated that microbial translocation in the context of chronic HIV infection can trigger systemic immune activation—a key predisposing factor in the development of AIDS-related non-Hodgkin lymphoma [8]. Persistent inflammatory status directly influences the lymphoid tissue by upregulating transforming growth factor (TGF) and triggering the production of collagen. These changes in the fibroblastic reticular network alter the structure and function of lymphoid tissue and reduce the number of naïve T cells [9]. Vaughan et al. analyzed the expression of TGF-β in HIV-associated DLBCL patients. Their study showed an inverse relationship between TGF-β1 and Treg expression of CD39 and Helios, which are markers associated with highly immunosuppressive regulatory T-cell phenotypes. This finding underscores the paradoxical role of elevated TGF-β1 in disrupting the immune microenvironment and impairing effective anti-tumor immune responses. Also, high TGF-β1 levels were positively correlated with neutrophil and monocyte counts, which are both indicators of systemic immune activation and might suggest a potential link to disease burden [10]. Continuous immune activation and inflammation alter the thymic function, which plays a key role in complete immune recovery. This induced and persistent activation of the thymus leads to inefficient thymopoiesis, atrophy, and fibrosis, with clonal exhaustion of T cells and aberrant development of regulatory T cells (Tregs), which creates an opportunistic environment for infections and inadequate control of HIV [11]. Studies have emphasized the significance of the thymus in supporting efficient gut mucosal defense. De Voeght et al. identified a connection between impaired thymic function, microbial translocation, and immune activation. Their findings suggested that enhancing thymic output could help mitigate altered immune activation resulting from HIV infection or HIV-induced microbial translocation [12]. Makgoeng et al., in their meta-analysis of prospective studies, emphasized the correlation between HIV infection, chronic inflammation, and immune activation, as reflected by elevated levels of immune stimulatory markers, and the increased risk of NHL development or progression [13].

### 2.2. Upregulation of miRNA-99 and miRNA-146a in HIV-Infected Macrophages

HIV-infected macrophages show elevated levels of miRNA-99, which inhibits cellular pathways responsible for HIV degradation. The persistence of infected macrophages leads to pyroptosis and chronic inflammation, driven by the release of tumor necrosis factor alpha (TNF-α), IL-6, and IL-1β cytokines. Another upregulated molecule in macrophages is miRNA-146a, which suppresses components of the innate immune response by downregulation of C–C Motif Chemokine Ligand 5 (CCL5). This contributes to diminished immune cell recruitment and promotes viral persistence [14]. However, a study based on the South African cohort of patients with DLBCL showed that low levels of pro-inflammatory (M1) macrophages were associated with poorer survival, while alternatively activated (M2) macrophage enrichment was linked to a better response to rituximab and did not predict survival. The relationship between macrophage phenotype and density within the tumor microenvironment and clinical outcomes in HIV-associated DLBCL is still insufficiently explored [15].

### 2.3. Suppression of Immune Responses Mediated by Indoleamine 2,3-Dioxygenase and NK Cell Receptors

Indoleamine 2,3-dioxygenase (IDO), an enzyme predominantly produced by macrophages and dendritic cells, is enhanced by Tat and Nef HIV proteins. Its elevated expression leads to depletion of Th17 cells, expansion of Treg cells, and reduced proliferation of cytotoxic T cells. The resulting immune suppression favors HIV to evade detection, simultaneously altering gut barrier integrity, which further expands susceptibility to infections [16,17,18]. Vaughan et al. analyzed the kynurenine/tryptophan ratio (KTR), which is a marker of IDO enzyme activity and immune suppression. They found levels were elevated above the normal range in nearly half of the patients (47.5%), with significantly higher levels in PLWHIV. KTR correlated with advanced disease stage, β2-microglobulin, and ferritin levels but showed no association with CD4 count, cytokines, or Treg numbers [10]. A strategy that could be used to counteract HIV-generated immune suppression and T-cell reduction is downregulation of IDO by using Epacadostat or 1-Methyl-Tryptophan (1-MT) as pharmacological inhibitors [19,20]. Another option is to block Tat and Nef HIV proteins that upregulate IDO, indirectly controlling IDO levels.

Natural killer cells (NK) are also affected by HIV infection, which reduces the expression of both NK receptor group 2 member D (NKG2D) and natural cytotoxicity receptors (NCRs). With reduced levels of these activating receptors, NK cells’ ability to recognize pre-cancerous tissues is altered, increasing cancer risk [21]. IL-15-centered therapy might represent an immunostimulatory strategy by boosting NK cell survival, proliferation, and tissue migration, which could help control HIV infection. However, IL-15 treatment requires further research to determine long-term benefits, safety, and possible interactions with other immune pathways [22]. Alternative strategies that have the potential to restore NK cell function include NKG2D agonists, which can upregulate NKG2D expression, or gene therapy, using CRISPR-based strategies to enhance NK cell receptor expression. A case report by Wang et al. described a 28-year-old man with HIV-associated DLBCL who was refractory to three lines of rituximab-based chemo-immunotherapy and radiotherapy. Surprisingly, the patient achieved and sustained a pathologically complete remission following treatment with haplotype-matched invariant NK (iNKT) cells in combination with anti-CD20 antibody. This outcome emphasizes the potential of iNKT cell-based immunotherapy as an intriguing strategy for the treatment of relapsed or refractory DLBCL, particularly in cases where standard therapies were unsuccessful [23].

### 2.4. Co-Infection with Oncogenic Virus

Beyond immune dysfunction, co-infection with oncogenic viruses also contributes to the development of HALs. A key example is the Epstein–Barr virus (EBV), a significant driver of lymphomagenesis through uncontrolled EBV-driven B cell proliferation that leads to abnormal activation and malignant growth of these cells [24]. EBV produces latent membrane protein 1 (LMP1) and EBV-encoded nuclear antigen 2 (EBNA2), which play a critical role in promoting lymphomagenesis. LMP1 imitates the physiological signaling of CD40 [25] and influences nuclear factor kappa–light–chain–enhancer of activated B cells (NF-kB) and Akt pathways [26,27], whilst EBNA2 complements these effects by enhancing pro-survival signals [28].

### 2.5. Tumor Immune Microenvironment Therapeutic Approaches

Overall, HIV straightforwardly impairs the immune system, causing aberrant cytokine secretion, immunosuppression, and damage to CD4^+^T cells and macrophages [29]. Patients with HIV face a substantially increased risk of developing lymphomas compared to those in the general population. The tumor immune microenvironment (TIME) in HALs indicates the interaction between tumor cells, immune cells, cytokines, and the surrounding extracellular matrix within a tumor. It shows how initially protective immune cells are reprogrammed by tumor signals and oncogenic viruses to support tumor growth, immune suppression, and metastasis. Tumor-associated macrophages (TAMs), NK cells, T cells, B cells, oncogenic viruses, and cytokines may play a dual role in TIME, finally promoting tumor development and progression [30,31]. TGF-β (Transforming Growth Factor Beta) might serve as an example by working as a tumor suppressor in early-stage of cancer, inhibiting uncontrolled cell growth and proliferation, and as a tumor promoter and immune suppressor in the late-stages of cancer by weakening NK cells’ tumor-killing ability, downregulating NKG2D expression and impairing their expertise in immunorecognition of tumor cells [32]. Consequently, cytokine therapy using TGF-β inhibitors might prevent immune exhaustion and maintain NK cells’ normal function.

Chantziou et al. studied the impact of HIV on the tumor microenvironment (TME) of classic HL and revealed an impaired immune response generated by this HIV and EBV co-infection. Reduced numbers of CD8^+^ T cells expressing PD-1 and TIGIT, downregulated T-cell receptor (TCR) signaling, increased numbers of CD155 high neoplastic cells, and upregulated extracellular matrix remodeling pathways were the major modifications depicted in this study. These impaired immune responses show the link between HIV infection and immune suppression, altered immune cell distribution, and structural remodeling of the TME—factors that may together contribute to diminished immune control and less favorable clinical trajectory [33].

**Figure 1:**
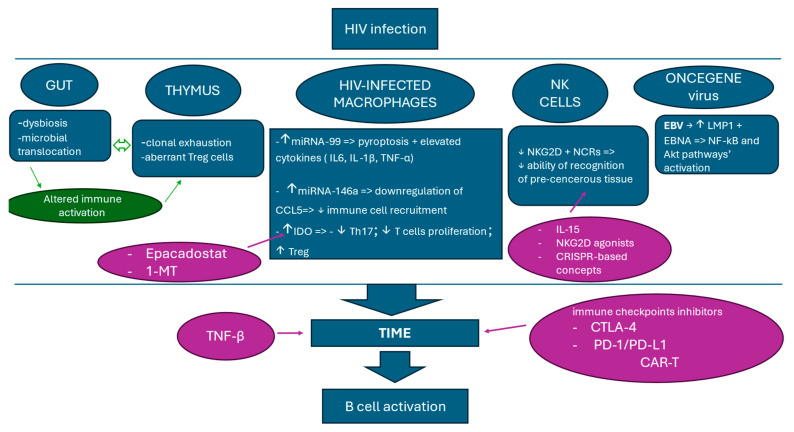
Chronic inflammation is seen as initiator of B cell activation via multiple indirect pathways (TIME). HIV infection targets primarily gut, whose disruptions lead to altered immune activation and collagen production. This structural damage influences the thymic function, causing inefficient T cell production and weakening immune response to HIV infection. Another mechanism involved in impaired immune cell recruitment and chronic inflammation is related to HIV-infected macrophages. Upregulated miRNA-99 and miRNA-146a cause elevated levels of tumor necrosis factor (TNF-α), IL-6, and IL-1β cytokines, pyroptosis, and suppressed immune cell recruitment. Additionally, IDO enzymes, enhanced by HIV proteins, deplete Th17 cells and expand Tregs, suppressing immunity. Its effect might be counteracted by IDO inhibitors (Epacadostat, 1-Methyl-Tryptophan (1-MT)). HIV infection also causes reduced NK cell function by downregulating NK receptor group 2 member D (NKG2D) and natural cytotoxicity receptors (NCRs). IL-15 therapy, NKG2D agonists, or CRISPR-based gene therapy may restore NK activity. Lastly, Epstein–Barr virus (EBV) co-infection increases lymphoma risk by upregulating latent membrane protein 1 (LMP1) and EBV-encoded nuclear antigen 2 (EBNA2) and activating nuclear factor kappa–light–chain–enhancer of activated B cells (NF-kB) and Akt pathways. Altogether, it creates a prone environment where immune cells are reprogrammed to promote tumor cell proliferation by hyperstimulation of the B cell tumor immune microenvironment (TIME). TGF-β inhibitors may prevent immune exhaustion, while immune checkpoint inhibitors (cytotoxic T-lymphocyte antigen-4 (CTLA-4), programmed death-1/programmed death ligand-1 (PD-1/PD-L1)) and chimeric Antigen Receptor T-cell (CAR-T) therapy offer potential treatments for HIV-associated lymphomas (HALs).

Reducing immune suppression in TIME by using immune checkpoint inhibitors serves as a promising therapeutic strategy. Cytotoxic T-lymphocyte antigen-4 (CTLA-4) is a T cell receptor that prevents excessive immune activation. CTLA-4 inhibitors, like Ipilimumab, can reactivate T-cell-mediated immune responses, attacking tumor cells more efficiently, whilst programmed death-1/programmed death ligand-1 (PD-1/PD-L1) inhibitors have a similar role in reducing immune suppression. These inhibitors can remove restrictions in the tumor microenvironment, reactivate T-cell-mediated immune responses, and boost HIV-specific immunity [34]. Chimeric Antigen Receptor T-cell therapy (CAR-T) is a type of adoptive immunotherapy based on genetically altering T cells to attack lymphoma cells and might have a positive impact in treating lymphomas. More data are required to evaluate the long-term effects and safety of this therapy [5] (Figure 1).

### 2.6. B Cell Activation and Expansion

Taken together, the effects of microbial translocation, thymic dysfunction, and affected T cell function lead to inflammation and abnormal activation of innate lymphoid cells, monocytes, and NK cells [35]. HIV-mediated immune dysfunction can induce chronic activation of B cells and may subsequently trigger lymphomagenesis [36]. In HIV patients, the immune system is in close contact with antigens from infections, HIV itself, or microbial translocation that, along with B-cell growth-promoting cytokines, IL-6 and IL-10, create a microenvironment that favors oligo- and polyclonal expansion of B lymphocytes [37]. The persistent stimulation and uncontrolled proliferation imply an elevated risk of acquiring dangerous genetic abnormalities. The most common genetic mutations found in HIV-infected patients with lymphomas are BCL-2, BCL-6, TP53, and MYC [38].

### 2.7. Immune Dysfunction Conditioned by Dysregulated BAFF Levels

B-cell activating factor (BAFF) is a signaling protein that plays a significant role in B-cell differentiation, survival, and antibody production. PLWHIV have elevated BAFF levels despite antiretroviral therapy (ART). The study by Doyon-Laliberte et al. emphasized how chronic BAFF stimulation downregulates the levels of NR4A1, NR4A3, CD83, and B regulatory cells (Bregs) in human precursor-like marginal zone B cells [39].

NR4A transcription factors relate to a family of NR4A1, NR4A2, and NR4A3 nuclear receptors that coordinate differentiation, proliferation, apoptosis, and inflammatory responses in immune cells, balancing immune suppression and activation. Their downregulation leads to a reduction in B-cell tolerance, improper regulation of immune responses, and enhanced B-cell exhaustion [40]. Along with the underexpression of CD83 and Breg depletion, they generate an environment susceptible to malignant transformation. Potential therapeutic strategies that might be used to target this mechanism are dihydroergotamine (DHE) by upregulating NR4As levels and Belimumab (Benlysta), which blocks BAFF and minimizes inflammatory overcharge [39].

### 2.8. Abnormal CD40L Incorporation in HIV and Its Consequences

Another mechanism involved in B cell activation is related to CD40L (CD40 Ligand), which is a costimulatory molecule expressed on the T cell’s surface. In normal conditions, CD40L binds to CD40 on B cells and activates them to generate high-affinity antibodies. In HIV patients, CD40L is incorporated into the virus particle, leading to CD40L-bearing HIV virions that can connect with CD40 on B cells and mimic physiological stimulation in an exaggerated way [41]. This hyperactivation of B cells affects the expression of activation-induced cytidine deaminase (AID), which is involved in somatic hypermutation and class switch recombination [42].

### 2.9. Proto-Oncogenes Mutations Caused by AID Overexpression

Overexpression of AID leads to point mutations, deletions, substitutions, and duplications in BCL-6, MYC, PIM1, and other key proto-oncogenes [42,43]. Moreover, AID induces chromosomal translocations that represent typical genetic alterations in DLBCL [44]. A common pattern of chromosomal translocation implicates the transfer of proto-oncogenes BCL-2, BCL-6, and MYC to a site near an immunoglobulin locus, specifically, the immunoglobulin heavy-chain (IgH) locus on chromosome 14. This modification indicates that the proto-oncogenes are controlled by IgH regulatory elements, leading to inappropriate overexpression [45,46]. BCL-2 translocation, reported as t (14;18), can generate elevated protein expression with anti-apoptotic action [47]. BCL-6 translocations have been noticed in two primary forms, either involving the immunoglobulin heavy-chain (IgH) locus on chromosome 14, known as t (3;14), or occurring at non-Ig loci—histone H4 [48]. MYC/IgH recombination, reported as t (8;14), is less common but noticed in BL, causing MYC overexpression with aggressive B cell proliferation [49]. These changes stimulate the development of B-cell lymphomas, especially BL and DLBCL.

### 2.10. Hyperactivation of B Cell Surface Markers

HIV-induced B cell activation generates an uncontrolled immunoglobulin release, with a lower response to antigenic stimulation and elevated secretion of IgM, IgG, and IgA [50]. Persistent activation causes alterations in surface markers of B cells, seen as enhanced expression of CD38, CD80, CD86, and CD95. CD80 and CD86 are costimulatory molecules that interact with T cells by activating them and initiating an immune response [51]. However, PLWHIV has higher baseline levels of CD86 and CD80, making B cells unable to respond properly and leading to a dysfunctional immune response [52]. On the other hand, elevated levels of CD38 suggest a more activated condition of B cells and, together with high CD95 levels, contribute to their increased susceptibility to apoptosis [53]. Hybel et al. further investigated and hypothesized a role of CD38 as a potential treatment target in lymphoma patients with HIV [54]. This combination of hyperactivation and increased apoptosis induces reduced antibody production, immune system exhaustion, and heightened vulnerability to infections in PLWHIV [51].

**Figure 2:**
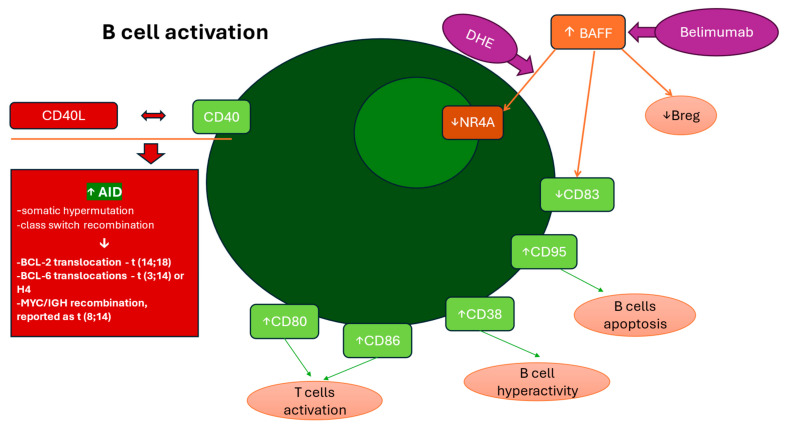
Chronic B cell activation—an increased risk of lymphomagenesis. B-cell activating factor (BAFF) overexpression in HIV patients disrupts B-cell regulation by downregulating NR4A1, NR4A3 transcription factors, CD83, and B regulatory cells (Bregs), contributing to immune exhaustion and malignancy. Targeted therapies like dihydroergotamine (DHE) (upregulating NR4As) and Belimumab (blocking BAFF) may help mitigate these effects. CD40 Ligand (CD40L) incorporation into HIV virions abnormally stimulates B cells, leading to activation-induced cytidine deaminase (AID) overexpression, which induces genetic mutations and chromosomal translocations (e.g., BCL-2 t(14;18), BCL-6 t(3;14), and MYC t(8;14)), promoting lymphoma development. Upregulated CD38, CD80, CD86, and CD95 drive B cell dysfunction, immune exhaustion, and apoptosis, weakening the immune system and increasing infection susceptibility.

To underline the fact that B cell hyperactivation in HIV infection may contribute to increased risk of AIDS-related lymphomas, Breen et al. investigated the correlation between two B-cell stimulatory molecules—soluble CD23 (sCD23) and IL-6 and lymphoma development. The results showed that soluble CD23 significantly increased in individuals who developed lymphoma, while IL-6 was categorically elevated among those who developed BL. These findings confirm that the pathogenesis of the Burkitt’s/small noncleaved cell (BL/SNC) subtype of AIDS-related lymphoma involves a separate pattern of B cell hyperactivation, differing from the immunological mechanisms driving other subtypes [55]. (Figure 2).

### 2.11. HIV Binding to B Cells Through Alternative Surface Molecules

HIV can interact directly with surface molecules on B lymphocytes in the absence of the CD4 receptor. The interaction between HIV and B cells happens through gp120, variable heavy chain 3 (VH3), C-type lectin receptor, and CD21. gp120 binds to B cells via membrane immunoglobulins, which belong to the VH3 family and are abundant on the surface of B cells [56]. Gp120 also binds to C-type lectin receptors and CD21, which are known as complement receptors (CR2). By binding to B cells via CD21, HIV transforms B cells into carriers and extracellular reservoirs of viral particles, facilitating the potential transmission of infectious viral particles to CD4+ T cells [57,58]. However, the loss of CD21 on B cells creates a distinct CD21 low subpopulation with signs of immune dysfunction in HIV. These dysfunctional B cells serve as a marker of disease progression by emphasizing immune exhaustion, hyperactivation, and improper immune response [59].

### 2.12. p17 Variants Induced PAR1/EGFR/PI3K/Akt Pathway Activation

HIV does not directly infect B cells, but its viral proteins and the resulting immune impairment contribute to an environment that promotes B cell activation, survival, and genomic instability, which are elements responsible for malignant transformation [60]. The HIV-1 matrix protein p17 increases the susceptibility of lymph nodes to lymphoma growth and metastatic spread by promoting a pro-lymphangiogenic microenvironment [61]. It is a 132-amino-acid protein encoded by the Gag gene and is crucial for viral assembly and release. Studies on p17 from human NHL identified two p17 variants (vp17s) promoting B-cell growth and Akt pathway activation. These variants feature extra amino acid insertions at positions 117–118 (NHL-a101 variant) or 125–126 (NHL-a102 variant) and are more prevalent in HIV-positive patients with NHL plasma. These modifications differentiate standard p17, known as refp17, from vp17s, which exhibit significant structural destabilization [62].

### 2.13. Pr55Gag Binding to PI (4,5) P2 at the Plasma Membrane Induce vp17s Release

Pr55Gag is a polyprotein localized on the plasma membrane and is essential for the production of p17, capsid protein (p24), and nucleocapsid protein (p7) [63]. Pr55Gag binds to phosphatidylinositol 4,5-bisphosphate (PI (4,5) P2), attaching itself to the inner leaflet of the plasma membrane. This binding generates conformational changes in Pr55Gag, assuring the accessibility of cellular aspartyl proteases to cleavage sites and releasing vp17 [64]. Vp17s release might result from an HIV protease-dependent mechanism or in an independent way, suggesting the implication of alternative pathways or cellular proteases in their secretion [60]. After secretion, vp17s interact with protease-activated receptor 1 (PAR1) and trigger activation of PAR1/EGFR/PI3K/Akt signaling pathway [65]. This cascade of events is associated with abnormal proliferation, increased cell survival, and tumor development, linking vp17s to lymphomagenesis in PLWHIV. The generation of antibodies or small molecules that target vp17s might block this pathway activation. An anti-p17 antibody gene transduction or small-molecule inhibitors that attach to the PI (4,5) P2 binding pocket of the viral protein can be used to block the interaction between Pr55Gag and PI (4,5) P2 [66,67]. This novel strategy would prevent p17 interaction with receptors and activation of harmful pathways.

### 2.14. The PAR1/EGFR/PI3K/Akt Pathway

PAR1 is a G-protein-coupled receptor on the cell surface. Once activated, it initiates downstream signaling pathways by recruiting and activating Gq proteins that trigger the activation of matrix metalloproteinases (MMPs). MMPs cleave pro-ligands of epidermal growth factor receptor (EGFR), and the active form of ligands bind and transactivate EGFR. Once transactivated, EGFR initiates the PI3K/Akt signaling pathway [65]. Activation of PI3K leads to phosphorylation of phosphatidylinositol-4,5-bisphosphate (PI (4,5) P2) and production of phosphatidylinositol-3,4,5-trisphosphate (PIP3). PIP3 is an anchor for Akt and its activator phosphoinositide-dependent kinase 1 (PDK1), whilst PDK1, together with mTORC2 phosphorylate Akt at two sites, Thr308 and Ser473, activating it. Once activated, the PI3K/Akt pathway modulates the activity of multiple molecules involved in crucial cellular processes, cell cycle, DNA repair, and proliferation [68]. Dysregulation of this pathway can lead to uncontrolled growth. MMPs represent a potential therapeutic target. Inhibition ensured by Batimastat and Ilomastat can reduce the effect of vp17s on the formation of B-cell colonies. MMP inhibitors might control cell growth and interfere with the PAR1/EGFR/PI3K/Akt pathway [65].

### 2.15. Akt Activation Effect on CDK1, Rb, p53, and Other Players in Cancer Cell Growth and Survival

Cyclin-dependent kinase 1 (CDK1), retinoblastoma protein (Rb), signal transducer, activator of transcription 1 (STAT1), protein tyrosine phosphatase 1B (PTP-1B), and tumor suppressor protein (p53) represent molecules modulated by Akt activation and involved in cell proliferation. These molecules are implicated in the regulation of cell cycle progression, DNA damage response, and survival. Consequently, Akt might be responsible for cancer cell survival by downregulating tumor suppressors, Rb and p53, and upregulating cell growth controllers—CDK1 and STAT1 [61,69,70,71,72].

### 2.16. PI3K Additional Pathways in Cancer Progression

PI3K involves additional pathways by interacting with mitogen-activated protein kinase 8 (MAPK8), activating Abelson tyrosine kinase 1 (ABL) and Ras-related C3 botulinum toxin substrate 1 (RAC1) [73,74,75]. It was found that vp17s modulate different signaling molecules, leading to lymphoma progression. Consequently, NHL-a101 activates checkpoint kinase 1 (CHEK1), which is involved in maintaining genome stability and DNA damage repair, and CHEK2 in combination with p53 and human mutS homolog 2 (hMSH2) [68,76]. It also activates CDK4 and JAK1 kinases, helping tumor-cell growth and immune destruction bypass [77,78]. NHL-a102 lacks significant modulation of these additional pathways. Differences in the vp17 activity emphasize the diverse ways by which HIV proteins contribute to lymphomagenesis and tumor progression.

### 2.17. AT20 Seen as a Target for Neutralizing Antibodies or Small Molecules

The hydrophobic cluster at amino terminal 20 (HC-AT20) is a specific hydrophobic region in p17 with two amino acid residues that are critical to maintaining its structural integrity. In vp17s, tryptophan at position 16 (Trp16) and tyrosine at position 29 (Tyr29) detach from the hydrophobic cluster and cause conformational changes with exposure of functional epitopes, making the interaction with PAR1 more available [79]. This specific region, also known as the AT20 loop, attaches to the C–X–C Motif Chemokine Receptor (CXCR1, CXCR2) on the cell membrane. These two G-protein-coupled receptors activate the G-protein complex, inducing endothelial cell proliferation and vessel remodeling by activating PI3K/Akt and mitogen-activated protein kinase kinases/extracellular signal-regulated kinases (MEK/ERK1/2) pathways. In this way, vp17s might exert angiogenic and lymphangiogenic activity due to interaction with both CXCR1 and CXCR2 [80].

A previously described mouse IgG anti-p17 monoclonal antibody (mAb), MBS-3, targeting a linear epitope (AA 9–18) within the AT20 functional domain, completely inhibited p17 binding to Raji cells. Another mAb, MK-18, which binds to the p17 C-terminal domain (AA 115–132), exhibited strong neutralizing effects, likely due to steric hindrance [81]. These findings indicate the potential for using antibodies to block the binding of the functional AT20 epitope to its receptors, CXCR1 and CXCR2, in order to prevent their pro-angiogenic and pro-lymphangiogenic effects. Additionally, they create opportunities for developing antibodies that could inhibit interaction with PAR-1, preventing B-cell clonogenic and oncogenic activities. Moreover, in cART-treated patients, the AT20-based therapeutic vaccine for HIV-1 has been shown to induce and maintain a durable, neutralizing antibody-mediated response against the functional epitope AT20 (AA 9–28), which is responsible for pro-angiogenic and pro-lymphangiogenic effects and has successfully completed phase I clinical trials [82,83]. A subsequent study demonstrated that these vaccine-induced anti-AT20 antibodies could neutralize the clonogenic activity associated with a vp17, in which Trp16 and Tyr29 were mutated to alanine. This AT20-KLH KLH (keyhole limpet hemocyanin) vaccination might affect the angiogenesis and clonogenicity in PLWHIV and is seen as an up-and-coming strategy in treating HAL [79].

### 2.18. Tat and Nef Implication in B-Cell Transformation via c-MYC

HIV-1 Tat protein exerts various influences on B cells. It upregulates cellular MYC (c-MYC) oncogene by binding to activator protein 1 (AP-1), JunB, and activating c-MYC promoter [84]. B cells exposed to Tat show increased rates of chromosomal alterations that modify the genome and predispose to errors [85]. It can spontaneously penetrate B cells and directly influence them. In the intracellular environment, Tat encourages MYC/IgH rearrangements, which juxtapose oncogene MYC with the IgH locus, leading to its unregulated expression [86]. Tat triggers the activation of the Akt/mTORC1 pathway and downregulates transcriptional repressors of activation-induced cytidine deaminase (AICDA), c-Myb and transcription factor (E2F8). These modifications enhance cell survival, genomic instability, and malignant proliferation of B cells [87]. Overall, the combined effects of c-MYC activation, chromosomal aberrations, and genomic instability strengthen B-cell transformation increasing the incidence of HALs.

**Figure 3:**
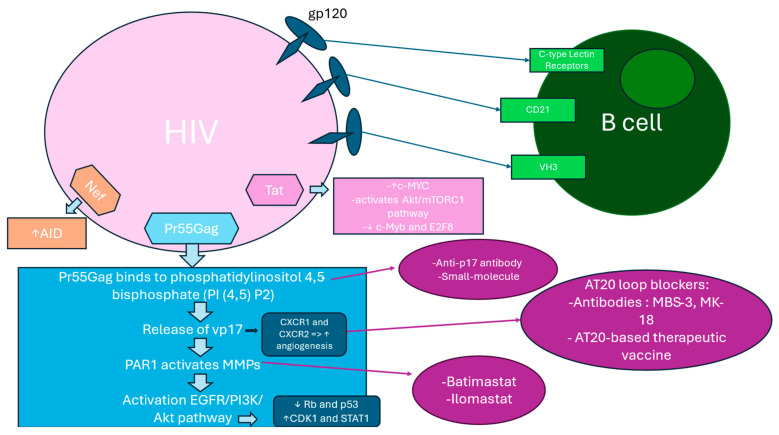
Direct interaction between HIV components and B cells. Vp17s activation of PAR1/EGFR/PI3K/Akt pathway. HIV directly interacts with B lymphocytes via gp120, variable heavy chain 3 (VH3), C-type lectin receptors, and CD21, allowing the virus to use B cells as viral reservoirs. Pr55Gag binds to PI (4,5) P2 on the inner plasma membrane, undergoes conformational changes, and exposes cleavage sites for aspartyl proteases, leading to vp17s release, which triggers protease-activated receptor 1 (PAR1) activation. This initiates a cascade of events in the PAR1/EGFR/PI3K/Akt pathway. Anti-p17 antibody gene transduction or small-molecule inhibitors targeting the PI (4,5) P2 binding pocket can block Pr55Gag interaction, preventing vp17-driven pathway activation. PAR1 triggers activation of matrix metalloproteinases (MMPs) that cleave pro-ligands of epidermal growth factor receptor (EGFR) and transactivate EGFR. Therapeutic strategies include inhibiting MMPs (e.g., Batimastat, Ilomastat) to prevent the PI3K/Akt pathway activation. The Akt pathway affects key cancer regulators (cyclin-dependent kinase 1 (CDK1), retinoblastoma protein (Rb), signal transducer and activator of transcription 1 (STAT1), and tumor suppressor protein (p53), further promoting B-cell survival and uncontrolled growth. This results in abnormal cell proliferation and B-cell oncogenic transformation. The AT20 hydrophobic cluster (HC-AT20) in vp17s increases the risk of angiogenesis and lymphomagenesis by interacting with PAR1 and C–X–C Motif Chemokine Receptor (CXCR1, CXCR2). Neutralizing antibodies (MBS-3, MK-18) and AT20-based vaccines have shown promise in blocking these interactions and limiting clonogenic activity in HIV-associated lymphomas (HALs). HIV Tat and Nef proteins contribute to B-cell transformation by upregulating c-MYC oncogene and AID, activating Akt/mTORC1 pathway and downregulating transcriptional repressors of activation-induced cytidine deaminase (AICDA)—c-Myb and E2F8.

HIV Nef protein plays an important role in promoting genomic instability and increasing the risk of B-cell transformation by upregulating AID expression. AID begins to target non-Ig loci, including proto-oncogenes like c-MYC, contributing to genomic instability and uncontrolled proliferation of B cells. Errors induced by AID are highly associated with the t (8;14) chromosomal translocation, a defining feature of BL. This translocation places c-MYC next to the IgH enhancer, resulting in uncontrolled c-MYC expression, which promotes B cell division and malignancy [88] (Figure 3).

## 3. Conclusions

During the last few years, studies have shown that PLWHIV has a higher risk of developing lymphomas despite the large use of cART. Lymphomagenesis might be driven by both direct and indirect pathways that contribute to the generation of TIME, genetic mutations, and proliferation of B cells, leading to the development of lymphomas. Moreover, these mechanisms highlight the complexity of lymphomagenesis in HIV-infected individuals, underscoring the need for a multifaceted approach to both prevention and treatment. Novel therapeutic approaches should be researched and further analyzed in large-scale clinical trials by targeting the mechanisms and molecules involved in HAL.

## Data Availability

No new data were created or analyzed in this study. Data sharing is not applicable to this article.

## Abbreviations

The following abbreviations are used in this manuscript:

1-MT	1-Methyl-Tryptophan
ABL	Abelson tyrosine kinase 1
AICDA	Activation-induced cytidine deaminase
AID	Activation-induced cytidine deaminase
AIDS	Acquired immune deficiency syndrome
AP-1	Activator protein 1
BL	Burkitt’s lymphoma
Breg	B regulatory cells
cART	Combined antiretroviral therapies
CAR-T	Chimeric Antigen Receptor T-cell therapy
CCL5	C–C Motif Chemokine Ligand 5
CD40L	CD40 Ligand
CDK1	Cyclin-dependent kinase 1
CHEK1	Checkpoint Kinase 1
c-MYC	Cellular MYC
CR2	Complement receptor 2
CTLA-4	Cytotoxic T-lymphocyte antigen-4
CXCR	C–X–C Motif Chemokine Receptor
DHE	Dihydroergotamine
DLBCL	Diffuse large B-cell lymphoma
E2F8	E2F transcription factor 8
EBNA2	EBV-encoded nuclear antigen 2
EBV	Epstein–Barr virus
EGFR	Epidermal growth factor receptor
HALs	HIV-associated lymphomas
HC-AT20	Hydrophobic cluster at amino terminal 20
HL	Hodgkin’s lymphoma
hMSH2	Human mutS homolog 2
IDO	Indoleamine 2,3-dioxygenase
IgH	Immunoglobulin heavy chain
IL	Interleukin
KLH	Keyhole limpet hemocyanin
KS	Kaposi’s sarcoma
LMP1	Latent membrane protein 1
mAb	Monoclonal antibody
MAPK8	Mitogen-activated protein kinase 8
MMPs	Matrix metalloproteinases
NCR	Natural cytotoxicity receptors
NF-kB	Nuclear factor kappa–light–chain–enhancer of activated B cells
NHL	Non-Hodgkin’s lymphoma
NK	Natural killer cells
NKG2D	NK receptor group 2 member D
PAR-1	Protease-activated receptor 1
PD-1	Programmed death-1
PDK1	Phosphoinositide-dependent kinase 1
PD-L1	Programmed death ligand-1
PI (4,5) P2	Phosphatidylinositol 4,5-bisphosphate
PIP3	Phosphatidylinositol-3,4,5-trisphosphate
PLWHIV	People living with HIV
PTP-1B	Protein tyrosine phosphatase 1B
RAC1	Ras-related C3 botulinum toxin substrate 1
Rb	Retinoblastoma protein
STAT1	Signal transducer and activator of transcription 1
TAMs	Tumor-associated macrophages
TGF	Transforming growth factor
TIME	Tumor immune microenvironment
TNF-α	Tumor necrosis factor
TNF-β	Transforming Growth Factor Beta
Tregs	Regulatory T cells
Trp16	Tryptophan at position 16
Tyr29	Tyrosine at position 29
VH3	Variable heavy chain 3
Vp17	p17 variants
